# Achieving circular-to-linear polarization conversion and beam deflection simultaneously using anisotropic coding metasurfaces

**DOI:** 10.1038/s41598-019-48812-y

**Published:** 2019-08-22

**Authors:** Yao Jing, Yongfeng Li, Jieqiu Zhang, Jiafu Wang, Maochang Feng, Tianshuo Qiu, He Wang, Yajuan Han, Hua Ma, Shaobo Qu

**Affiliations:** 1 Department of Basic Sciences, Air Force Engineering University, Xi’an, 710051 People’s Republic of China; 2 School of Physics and Optoelectronic Engineering, Xidian University, Xi’an, 710071 People’s Republic of China

**Keywords:** Materials science, Physics

## Abstract

An anisotropic coding metasurface (CM) is proposed for achieving circular-to-linear polarization conversion and beam deflection. Different phase coding consequences were independently achieved for two orthogonal linear polarized (LP) waves. Thus by elaborately designing coding sequences of the metasurfaces, different functions can be achieved, respectively for waves polarized along two orthogonal directions. More importantly, for circularly polarized (CP) wave, anisotropic CM can achieve circular-to-linear polarization conversion and beam deflection simultaneously. As a proof, a 1-bit anisotropic CM with 0101…/0101… and 0000…/1111… coding sequences respectively for two orthogonal LP waves was designed. The simulation results indicated that the incident CP wave is deflected into two *x*-polarized waves in *x*-*o*-*z* plane and two *y*-polarized waves in *y*-*o*-*z* plane. Both the simulation and experimental results verify the circular-to-linear polarization conversion performance of the anisotropic coding metasurfaces. The proposed anisotropic coding metasurfaces have the potential for the applications of multifunctional devices.

## Introduction

Since Yu *et al*. put forth the generalized Snell’s laws^1^, artificial metasurfaces composed of subwavelength structures have proven to be very effective for controlling electromagnetic (EM) waves^2–7^. Due to its low loss and thin thickness, metasurfaces is widely employed in the microwave^8–10^, terahertz^3,11^ and visible frequencies^12^, and its applications in many fields have resulted in quite good effects, such as stealth technology^13–16^ antenna technology^17–19^ and holographic technology^20,21^.

In recently year, coding metasurface (CM) was proposed^22^, which offers a new developing direction for metasurface. According to this concept, the design of metasurface is effectively combined with the binary codes. By designing different coding sequences, a variety of functions can be realized like anomalous reflection^7,23^, generation of orbital angular momentum^24^ and diffusion scattering^25,26^ of EM waves. This concept can be extended to higher bits for more flexible control of EM waves.

Not long ago, the concept of anisotropic CM has been proposed^27–29^, which can achieve two separate responses for orthogonal polarization EM waves. Because of special function of anisotropic CM, it is widely used in the development of dual-functional devices^30–32^. In addition, the anisotropic CM can not only realize anomalous reflections but also linear-to-circular polarization conversion. However, there are few studies on circular-to-linear polarization conversion and beam deflection by anisotropic CM. In this paper, we demonstrate the ability of anisotropic metasurfaces to transform circularly polarized (CP) waves into linear polarized (LP) waves and achieve beam deflection simultaneously. As an example, a 1-bit anisotropic CM with different coding sequences for *x*- and *y*-polarized incidence waves was designed. Both the simulation and experimental results support our prediction and demonstrate the circular-to-linear polarization conversion function of the anisotropic CMs.

## Results

### Operating principle and theoretical analysis

**Figure 1:**
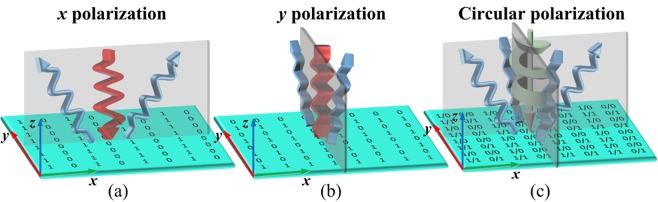
Schematics of the proposed anisotropic CM illuminated by different polarized wave. (**a**) The incident *x*-polarization wave is reflected into two equal waves along *x* direction. (**b**) The incident *y*-polarization wave is reflected into two equal waves along *y* direction. (**c**) The incident CP wave is split into four symmetrical waves.

In order to clarify multifunctionality of anisotropic CMs and their ability to transform CP waves into LP waves, we expound the principle by a simple 1-bit anisotropic CM. For the 1-bit anisotropic CM, coding sequences are composed of four basic encodings [0/0, 0/1, 1/0, 1/1]. The digit code before ‘/’ represents the phase state of the unit cell under *x*-polarization, while the digit code after ‘/’ represents the phase state of the unit cell under *y*-polarization. For a CM which is encoded with coding sequence [0,1;0,1], the incident *x*-polarization wave is reflected into two equal waves along *x* direction (Fig. 1(a)). Similarly, if the CM is encoded with coding sequence [0,0;1,1], the incident *y*-polarization wave is reflected into two equal waves along *y* direction (Fig. 1(b)). As it is known to all, a CP wave can be decomposed into two orthogonal LP waves with same amplitude and phase difference is 90°. Therefore, when anisotropic CM is illuminated by CP wave, the CP wave is transformed into LP wave and achieving beam deflection simultaneously, as shown in Fig. 1(c).

To mathematically describe the anisotropic CM, a tensor ${\overline{{\rm{R}}}}_{mn}$ is used to express the reflection coefficient of a unit cell indicated below:

1 $${\bar{{\rm{R}}}}_{mn}=[\begin{array}{cc}\hat{x}{R}_{mn}^{x} & 0\\ 0 & \hat{y}{R}_{mn}^{y}\end{array}]$$

where ${R}_{mn}^{x}$ and ${R}_{mn}^{y}$ denote reflection coefficients under *x*- and *y*-polarizations, respectively. For isotropic unit cells, ${R}_{mn}^{x}$ = ${R}_{mn}^{y}$; for anisotropic unit cells, the two reflection coefficients are different.

The anomalous reflection angle (*θ*, *φ*) can be obtained from theory of beam superposition of array antenna^22^, and the formulas is as follows:

2 $${\theta }={\sin }^{-1}(\lambda \sqrt{\frac{1}{{{L}}_{x}^{2}}+\frac{1}{{L}_{y}^{2}}})$$

3 $${\phi }=\pm {\tan }^{-1}\frac{{D}_{x}}{{D}_{y}},{\phi }=\pi \pm {\tan }^{-1}\frac{{D}_{x}}{{D}_{y}}$$

where 𝜆 is the wavelength of the free-space, and the lengths of one period of gradient phase distribution along *x*- and *y*- directions are marked by *D*_*x*_ and *D*_*y*_.

### Numerical simulation

Two types of unit cells, an anisotropic ‘split ring’-shaped and an isotropic ‘Crusades’-shaped metallic pattern are designed for anisotropic CM. Both unit cells have the same period *a* = 5.2 mm and thickness *d* = 3 mm.

**Figure 2:**
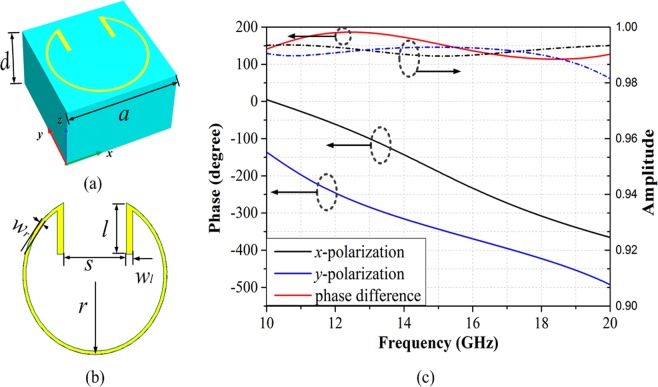
Geometries of the ‘split ring’ pattern. (**a**) The full view of the unit cell. (**b**) The metal ‘split ring’ pattern. (**c**) The simulated results of the reflection phases and amplitudes.

The ‘split ring’ pattern is used as anisotropic unit cell (Fig. 2). The top is metal ‘split ring’, and then dielectric substrates (*ε*_*r*_ = 2.65, tan *δ* = 0.001), the below is metal backboard. The other geometrical parameters are *r* = 2.3 mm, *w*_*r*_ = 0.1 mm, *s* = 2 mm, *l* = 1.48 mm, *w*_*l*_ = 0.2 mm. The reflected phases and amplitudes of the ‘split ring’ pattern under incident *x*-polarized and *y*-polarized wave are shown in Fig. 2(c). The phase difference between the two is about 180°, and the reflective amplitudes are more than 98%. Therefore, it is treated as ‘1’ and ‘0’ numeric state under *x*- and *y*-polarizations respectively.

**Figure 3:**
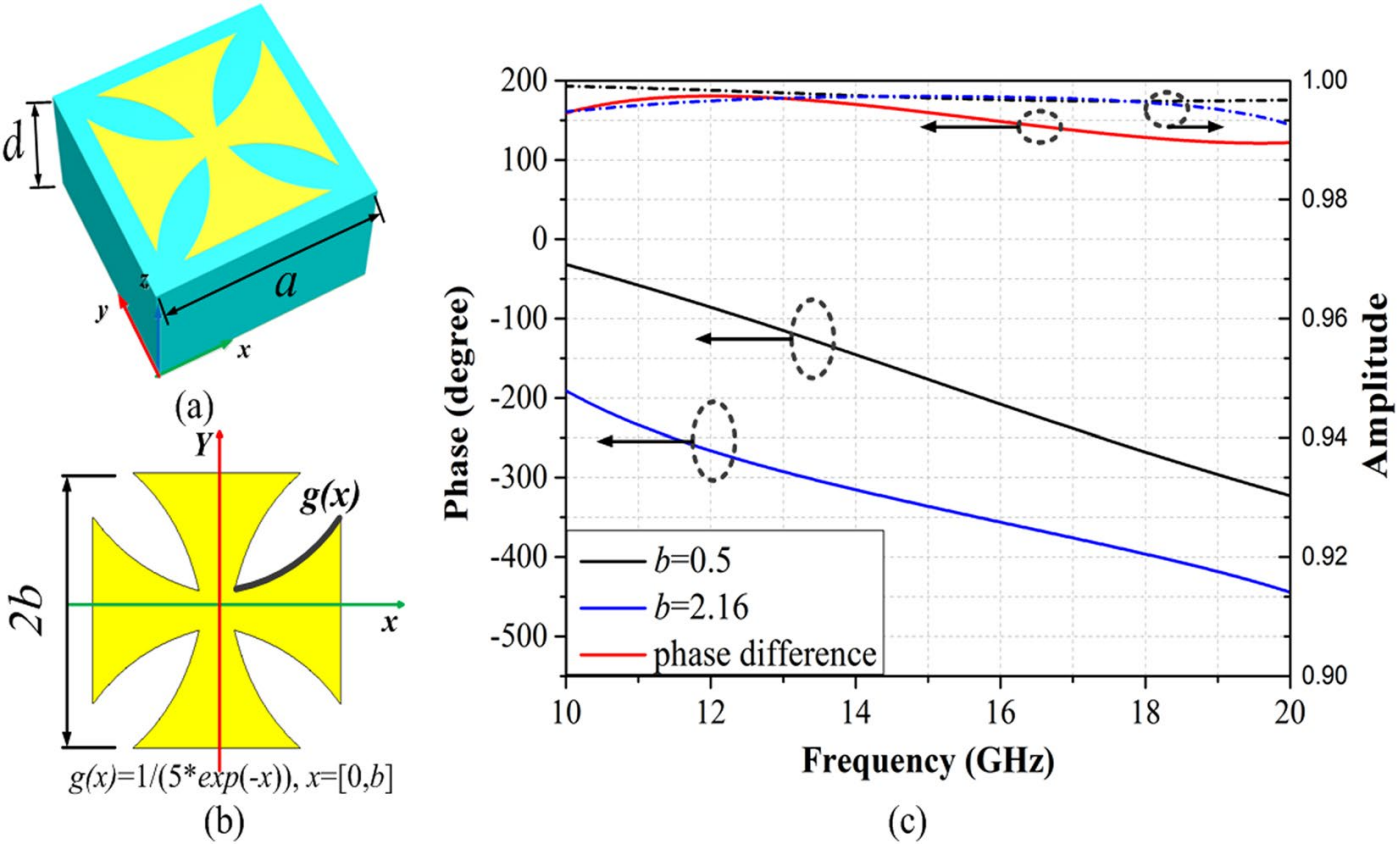
Geometries of the ‘Crusades’ pattern. (**a**) The full view of the unit cell. (**b**) The metal ‘Crusades’ pattern. (**c**) The simulated results of the reflection phases and amplitudes for the isotropic unit cell.

**Figure 4:**
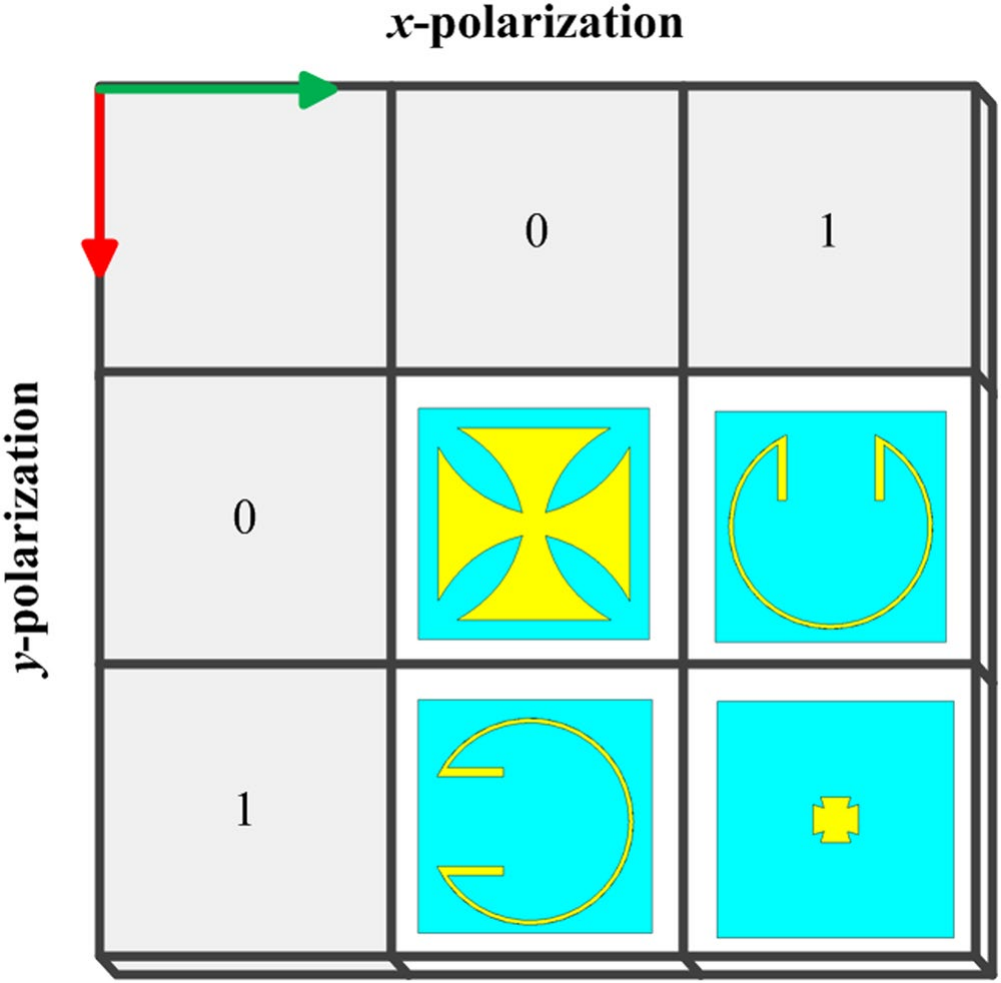
The four basic structures of 1-bit anisotropic CM.

The ‘Crusades’ pattern is used as isotropic unit cell (Fig. 3). The top is metal ‘Crusades’, the other two layers are the same as the anisotropic unit cell. By optimizing design, the ‘Crusades’-shaped metallic pattern with *b* = 0.5 mm and *b* = 2.16 mm are treated as ‘1’ and ‘0’ numeric state respectively and the corresponding reflection phases and amplitudes are shown in Fig. 3(c). The four basic structures of 1-bit anisotropic CM are shown in the Fig. 4.

In this paper, two different coding sequences are presented to demonstrate the special ability of anisotropic metasurface. The first coding sequence is composed of a periodic coding matrix *C*_1_:

$${C}_{1}=(\begin{array}{cc}1/0 & 0/0\\ 1/1 & 0/1\end{array})$$

**Figure 5:**
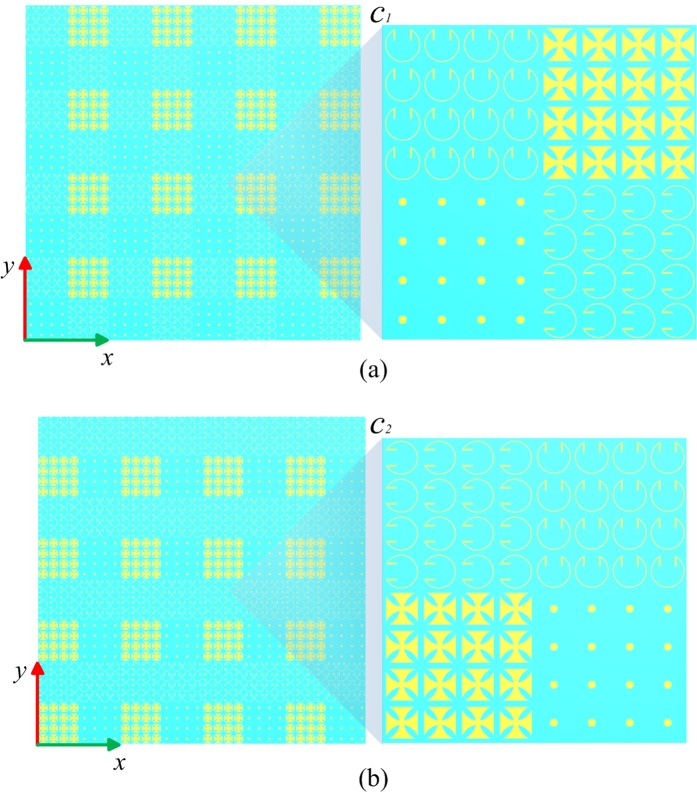
The completed views and enlarged views of the designed anisotropic CMs. (**a**) The anisotropic CM with matrix *C*_1_. (**b**) The anisotropic CM with matrix *C*_2_.

**Figure 6:**
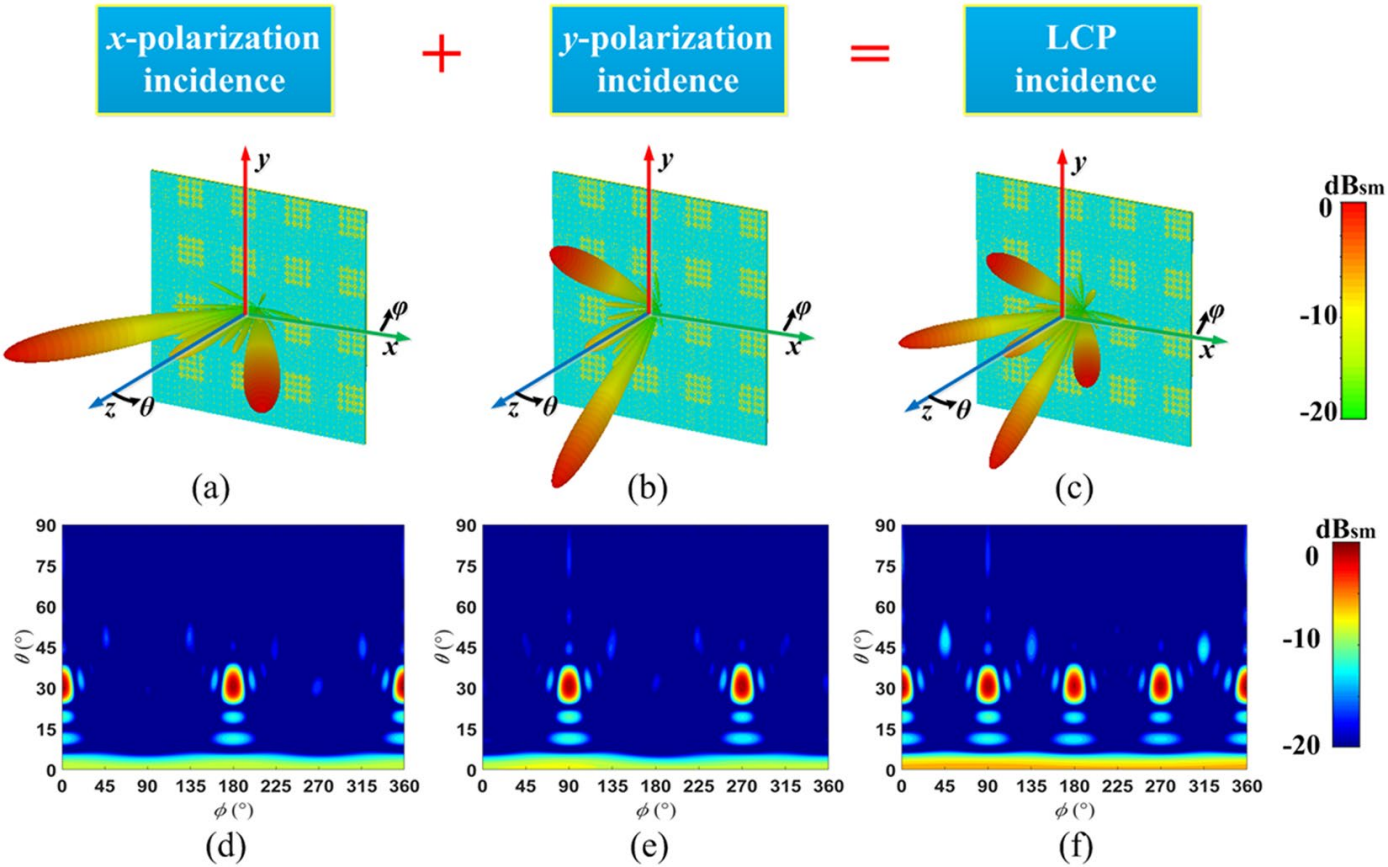
The simulation results of the anisotropic CM with matrix *C*_1_ under incidence of *x*-polarization, *y*-polarization and LCP waves at 14 GHz. (**a**–**c**) 3D and (**d**–**f**) 2D far-field scattering pattern.

**Figure 7:**
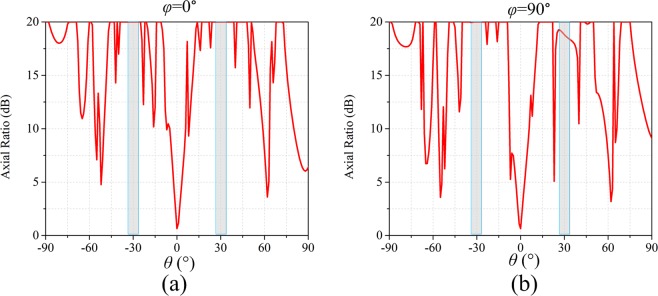
The axial ratio of the reflected wave in *φ* = 0° (**a**) and *φ* = 90° (**b**) cutting plane for the anisotropic CM with matrix *C*_1_ under the LCP incidence at 14 GHz.

The completed view of the anisotropic CM with matrix *C*_1_ is show in Fig. 5(a). The three-dimensional (3D) and two-dimensional (2D) far-field scattering patterns of anisotropic CM with matrix *C*_1_ under incident *x*-polarized wave at 14 GHz are show in Fig. 6(a,d) respectively. The simulated results show that the incident x-polarized wave is reflected into two symmetrical waves in *φ* = 0° cutting plane, and *θ* = 31.5°. The theoretical deviation angle is calculated as 31°, which is consistent with the numerical simulation. For the *y*-polarization, the incident wave is reflected into two symmetrical waves in *φ* = 90° plane, and the deviation angle *θ* = 31.5°, as shown in Fig. 6(b,e). So far we have proved the anisotropic properties of the designed CM. For the left-handed circularly polarized (LCP) incident wave, the wave is deflected to four symmetrical waves (*φ* = 0°, 90°, 180°, 270°, *θ* = 31°), as shown in Fig. 6(c,f). The simulated results indicates that the reflection characteristics of the anisotropic CM under CP wave incidence simultaneously possesses the both reflection characteristics under two orthogonal LP waves. To further verify the polarization characteristics of reflected beam, the axial ratio are analyzed. As shown in Fig. (7), two cutting planes *φ* = 0°, 90° are selected, and the axial ratio of the reflected waves (*θ* = ±31°) exceed 18 dB. Therefore the CP incident wave is converted to LP wave as expected.

To test this further, a periodic coding matrix *C*_2_ is designed:

$${C}_{2}=(\begin{array}{cc}0/1 & 1/0\\ 0/0 & 1/1\end{array})$$

**Figure 8:**
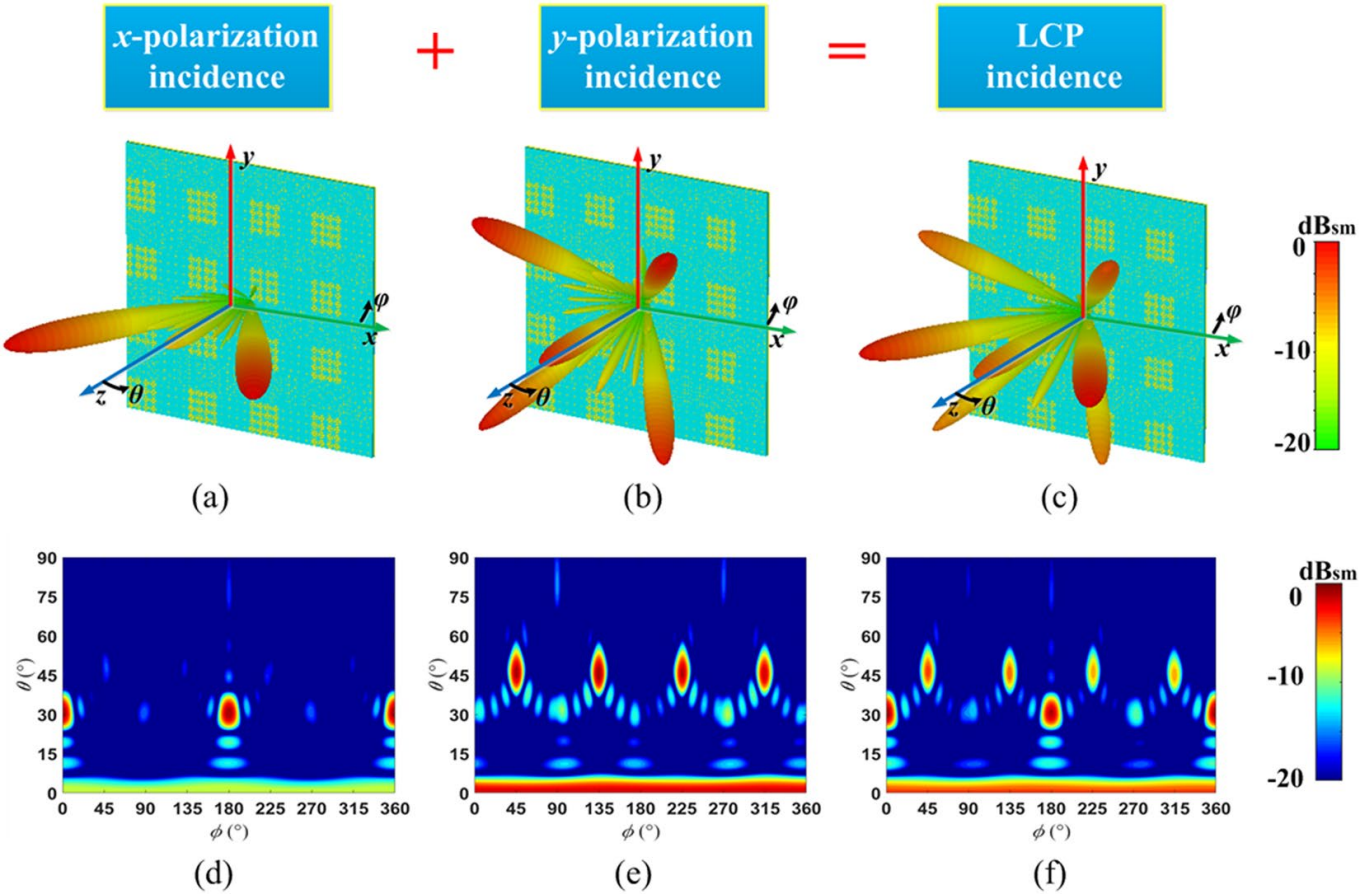
The simulation results of the anisotropic CM with matrix *C*_2_ under incidence of *x*-polarization, *y*-polarization and LCP waves at 14 GHz. (**a**–**c**) 3D and (**d**–**f**) 2D far-field scattering pattern.

**Figure 9:**
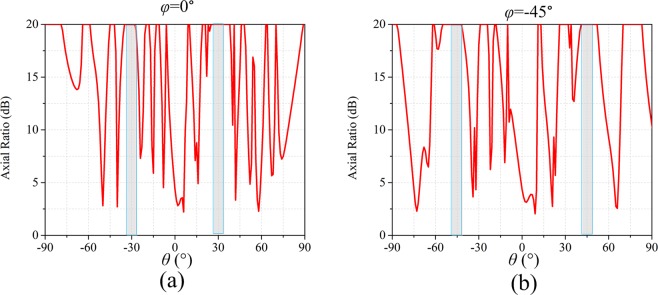
The axial ratio of the reflected wave in *φ* = 0° (**a**) and *φ* = −45° (**b**) cutting plane for the anisotropic CM with matrix *C*_2_ under the LCP incidence at 14 GHz.

The completed view of the anisotropic CM with matrix *C*_2_ is show in Fig. 5(b). The 3D and 2D far-field scattering patterns of anisotropic CM with matrix *C*_2_ under incident *x*-, *y*-polarized and LCP wave at 14 GHz are show in Fig. 8. For the *x*-polarization (Fig. 8(a,d)), the incident wave is deflected to two symmetrical waves in *φ* = 0° cutting plane (*θ* = 31°). For the *y*-polarization (Fig. 8(b,e)), the incident wave is deflected into four waves in diagonal direction (*φ* = 45°, 135°, 225°, 315°, *θ* = 46.75°). Whereas for LCP wave, the wave is split into six directions as anticipated. Similarly, the axial ratio of reflected waves are analyzed, two cutting planes *φ* = 0°, −45° are selected, and the axial ratio of the reflected waves (*φ* = 0°, *θ* = ±31°; *φ* = −45°, *θ* = ±46.75°) are all above 20 dB, as shown in Fig. 9.

The results of these simulations are consistent with the theoretic analysis, it indicates that anisotropic CMs can convert the CP wave into LP wave and achieve beam deflection simultaneously.

## Experiment

**Figure 10:**
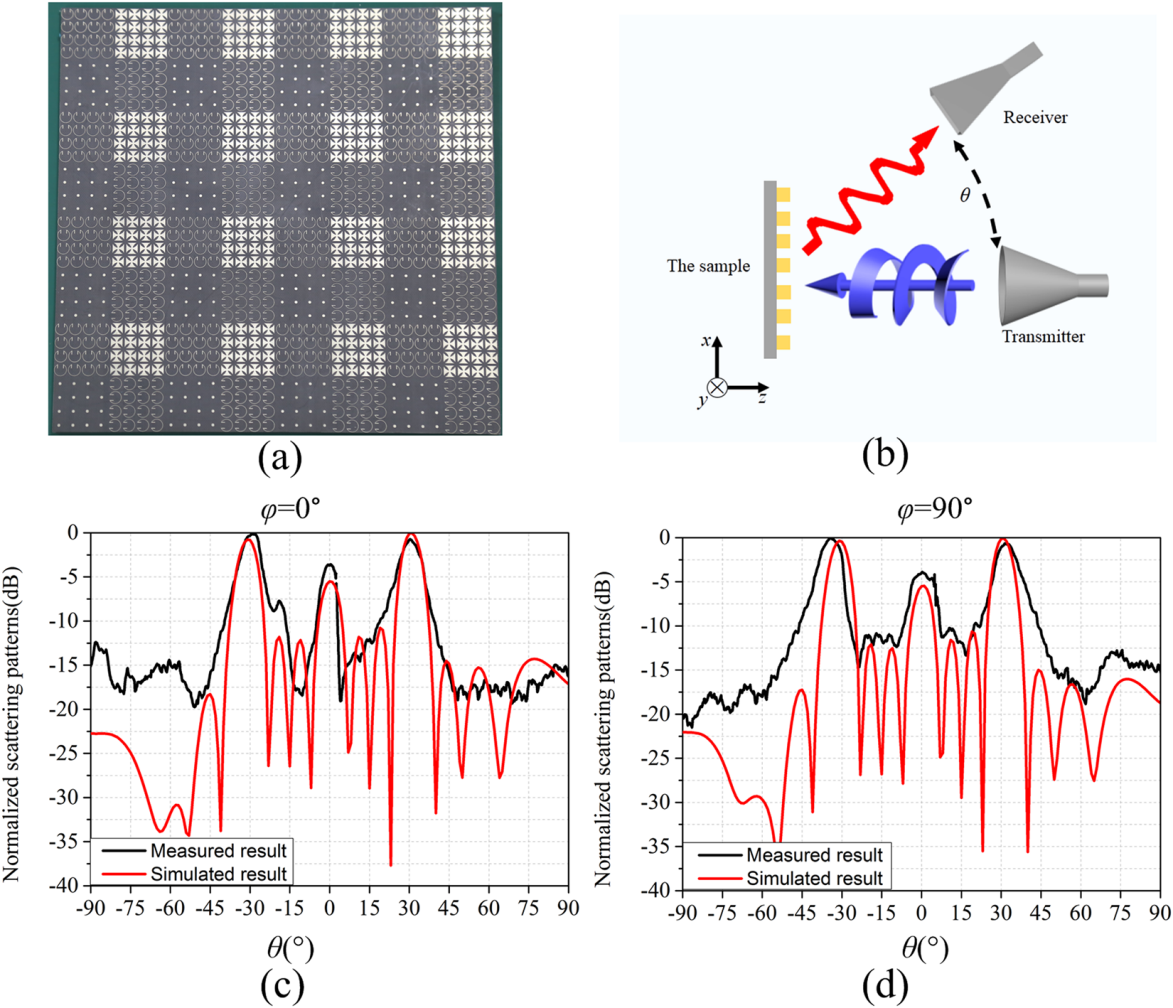
Experiments and results. (**a**) Photograph of the sample. (**b**) the sketch maps of the test setup. The comparisons of simulated and measured results of the anisotropic CM with matrix *C*_1_ at the azimuth angle *φ* = 0° (**c**) and *φ* = 90° (**d**).

To validate the performance of the designed anisotropic CMs, one sample (as shown in Fig. 10(a)) is fabricated, which corresponds to the coding matrix *C*_1_. The measurement was carried out in a microwave anechoic chamber, and the sketch maps of the test setup is given in Fig. 10(b). The measured 2D far-field scattering patterns of the anisotropic CM with matrix *C*_1_ at 14 GHz are show in Fig. 10. The results show that the measured deviation angle is accordance with the simulated result roughly, which demonstrates that anisotropic CMs can convert CP incident wave into LP wave and achieve beam deflection simultaneously.

## Conclusions

In conclusion, we have proposed an anisotropic CM, which can achieve circular-to-linear polarization conversion and beam deflection simultaneously. Due to the characteristic of anisotropic, the coding sequences depend on the EM wave’s states of polarization, and the manipulation of EM waves becomes more flexible. In other word, the same metasurfaces can achieve different functions under the different polarized incident waves. Moreover, by analyzing the axial ratio of reflected EM waves, it is found that the anisotropic CMs can convert CP wave into LP wave. Both the simulated and measured results indicate that anisotropic CM can achieve circular-to-linear polarization conversion and beam deflection simultaneously. The proposed anisotropic CMs have the potential for the applications of multifunctional devices.

## Methods

### Simulations

EM simulations were simulated with CST Microwave Studio. The reflected amplitudes and phases of unit cells were simulated using the Frequency domain solver. The unit cell boundary was set for both the *x* and *y* directions, and the open at space boundary for the *z* directions. The full model was calculated using the Time domain solver with open boundary conditions along all directions.

### Measurements

The measurement was made in a microwavechamber. A CP horn antenna served as transmitter, and maintained 2 m distance from the sample. Both the sample and the CP horn were bolted to the revolving stage, which could rotate 360°. In addition, a LP horn antenna was used as the receiving antenna to receive the scattering fields.
